# Astroglial changes in the zona incerta in response to motor cortex stimulation in a rat model of chronic neuropathy

**DOI:** 10.1038/s41598-020-57797-y

**Published:** 2020-01-22

**Authors:** Myeounghoon Cha, Kyung Hee Lee, Bae Hwan Lee

**Affiliations:** 1 Department of Physiology, Yonsei University College of Medicine, Seoul, 03722 Republic of Korea; 2 Department of Dental Hygiene, Division of Health Science, Dongseo University, Busan, 47011 Republic of Korea; 3 Brain Korea 21 PLUS Project for Medical Science, Brain Research Institute, Epilepsy Research Institute, Yonsei University College of Medicine, Seoul, 03722 Republic of Korea

**Keywords:** Neuroscience, Physiology

## Abstract

Although astrocytes are known to regulate synaptic transmission and affect new memory formation by influencing long-term potentiation and functional synaptic plasticity, their role in pain modulation is poorly understood. Motor cortex stimulation (MCS) has been used to reduce neuropathic pain through the incertothalamic pathway, including the primary motor cortex (M1) and the zona incerta (ZI). However, there has been no in-depth study of these modulatory effects and region-specific changes in neural plasticity. In this study, we investigated the effects of MCS-induced pain modulation as well as the relationship between the ZI neuroplasticity and MCS-induced pain alleviation in neuropathic pain (NP). MCS-induced threshold changes were evaluated after daily MCS. Then, the morphological changes of glial cells were compared by tissue staining. In order to quantify the neuroplasticity, MAP2, PSD95, and synapsin in the ZI and M1 were measured and analyzed with western blot. In behavioral test, repetitive MCS reduced NP in nerve-injured rats. We also observed recovered GFAP expression in the NP with MCS rats. In the NP with sham MCS rats, increased CD68 level was observed. In the NP with MCS group, increased mGluR1 expression was observed. Analysis of synaptogenesis-related molecules in the M1 and ZI revealed that synaptic changes occured in the M1, and increased astrocytes in the ZI were more closely associated with pain alleviation after MCS. Our findings suggest that MCS may modulate the astrocyte activities in the ZI and synaptic changes in the M1. Our results may provide new insight into the important and numerous roles of astrocytes in the formation and function.

## Introduction

Chronic neuropathic pain (NP) is the result of primary lesion in peripheral nerve and/or central nervous system (CNS) dysfunction in the absence of nociceptor stimulation^1^. This multidimensional clinical entity is mediated by various pathophysiological mechanisms, making its treatment difficult^2,3^. Neuropathic pain refractory to medication has been treated with invasive methods, such as selective lesioning and electrical stimulation of the central or peripheral nervous system^4^.

MCS was initially applied to central pain secondary to thalamic stroke^5,6^. Over time, its usage expanded to various types of chronic pain. Repetitive MCS relieves approximately 45 to 75% of pain^7,8^, making it a viable option for patients with severe drug-refractory symptoms. Motor cortex stimulation (MCS) treatment was first used in the early 1990s to treat a patient with chronic, drug-resistant NP^9^. Despite the clinical use of MCS for pain reduction, the mechanisms underlying its effects remain unclear. The incertothalamic pathway was recently described as a novel system for regulating nociceptive processing in the thalamus^10,11^. In this pathway, the zona incerta (ZI) inhibits the flow of nociceptive and somatosensory information in the posterior thalamus (Po), and this is mediated by the cholinergic system^10,12^.

Modulation of the ZI activity by repeated MCS was shown to be effective in ameliorating chronic NP^10^. Increased spontaneous and evoked activity in the ZI is causally related to repetitive MCS^10^. These findings strongly implicate the ZI as a site of maladaptive plasticity in chronic pain modulation. Understanding MCS-induced plasticity is of fundamental neurobiological importance, and is probably a requirement for developing effective strategies to promote recovery following brain damage^13^. However, little is known about MCS-induced neuronal changes in pain modulation.

The aim of this study was to explore how MCS regulates synaptic plasticity in the ZI. In particular, we focused on the relationship between pain modulation after MCS and neuronal plastic changes in the ZI, which has been associated with astrocyte-induced synapse modulation. Using a rat model of chronic NP, we compared neuronal changes after MCS and measured various markers of astrocyte plasticity in the ZI. Our findings suggest that MCS may modulate astrocyte activities in the ZI and synaptic changes in the M1. Furthermore, these results clarify the mechanism of MCS-induced analgesic effects in the setting of chronic NP.

## Results

### Development of NP and effects of MCS

**Figure 1:**
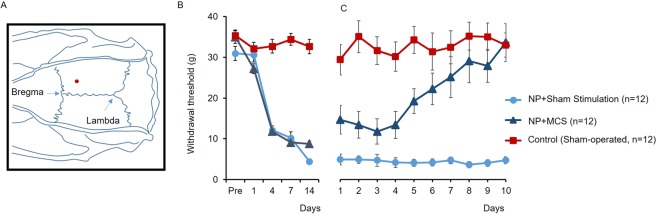
Identification of MCS site and mechanical threshold changes. (**A**) MCS electrode position. Platinum silver electrodes were placed stereotaxically over the primary motor cortex (M1) area contralateral to the injured hind paw; the location was defined by its coordinates with respect to bregma (red dot). (**B**) Changes in mechanical allodynia after nerve injury. On each test day (1, 4, 7, and 14 days after surgery), nerve-injured rats showed a significantly decreased mechanical withdrawal threshold compared to sham-operated group (**C**). Mechanical threshold changes between groups were compared in daily post-MCS session.

We previously reported that rats with peripheral nerve injury develop mechanical allodynia of the hind paw^14^. Consistent with this, we measured a significant reduction in the mechanical withdrawal threshold of the hind paw within 14 days of nerve injury. As shown in Fig. 1B, ipsilateral hind paw mechanical thresholds gradually decreased: 32.48 ± 1.66 g (pre), 28.89 ± 1.46 g (Day 1), 11.96 ± 0.97 g (Day 4), 9.64 ± 1.11 g (Day 7) and 4.61 ± 0.47 g (day 14). There was no significant decrease in sham animals (from 35.36 ± 1.36 g to 32.65 ± 0.47 g, *p* < 0.01 vs. NP rats). Importantly, repetitive MCS treatment reduced nerve injury-induced pain in rats. The withdrawal threshold of NP rats gradually increased following repetitive stimulation of the M1 cortex (*p* < 0.05 vs. NP + Sham stim).

### Astrocytes in ZI

**Figure 2:**
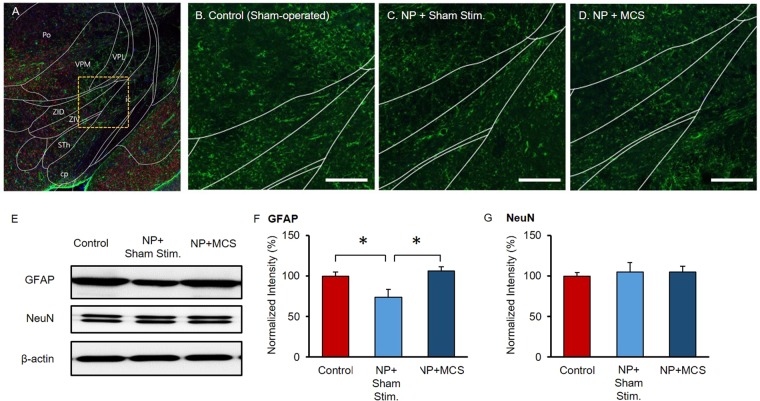
Astrocyte expression in the ZI. (**A**) Location of the ZI in the subthalamus. Box indicates the location of the ZI. (**B**) Astrocytes (GFAP, green) in the ZI of a control rat. (**C**) Astrocytes in the ZI of an NP rat 24 days after nerve injury, showing decreased GFAP expression compared to the control. (**D**) After 10 days of MCS sessions, GFAP expression levels in the ZI were compared. Repetitive M1 stimulation contributed to the restoration of GFAP expression in the ZI. (Scale bars = 200 µm.) (**E**) GFAP expression was compared among groups. Activity was normalized to the protein concentration. (**F,G**) Expression levels of GFAP and NeuN are shown as % changes compared to the control rats. Error bars indicate the standard errors calculated from three independent experiments. After nerve injury, GFAP protein levels were significantly reduced in the ZI. Following repetitive MCS, GFAP protein expression increased *(p* < 0.05). However, NeuN levels did not significantly change after MCS. Punctured ZI tissues were subjected to western blotting with GFAP or NeuN antibodies. Full-length blots are presented in Supplementary Fig. S1.

We observed astrocytes and neurons in the ZI area from −3.3 to −4.0 mm of the bregma. Figure 2A depicts the ZI area, and Fig. 2B–D show representative examples of astrocytes in different groups. A previous study reported pain-related neural activity in the dorsal and ventrolateral ZI^10^. We observed lower the ZI glial fibrillary acidic protein (GFAP) expression in NP + Sham stim. rats than in the control rats. Following repetitive MCS, GFAP expression in the ZI was increased compared to NP + Sham stim. group. We also performed western blotting to quantify GFAP expression levels in each group. Figure 2E shows a comparison of neuron-specific protein expressions. GFAP in the ZI significantly decreased after nerve injury (control, 100 ± 5.1; NP + Sham stim., 73.63 ± 9.69; NP + MCS, 105.93 ± 5.67; Fig. 2F). We quantified NeuN expression in the ZI region. However, the regional and cellular distributions of NeuN did not differ between the control and NP rats (control, 100 ± 4.5; NP + Sham stim., 105.03 ± 11.62; NP + MCS, 104.91 ± 7.2; Fig. 2G).

### Effect of MCS on synaptic plasticity

**Figure 3:**
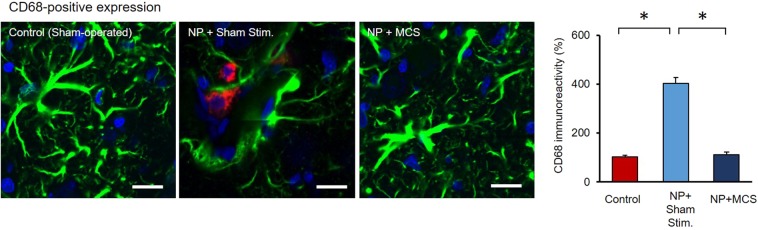
CD68 expression in the ZI of the rats. In order to identify protein expression by microglia, brain slices were labeled with CD68 (red), GFAP (green) antibodies, and DAPI (blue). CD68-positive signals were compared in each group. NP + MCS rats had no effect on the morphological change in CD68-positive signal. Frequent co-occurrence of astrocytes and microglial cells was observed in NP + Sham stim. rats. (Scale bars = 20 µm).

Astrocytes and microglia are actively involved in synaptic plasticity. Microglia are a type of neuroglia (glial cells) that are resident macrophage cells in the CNS. In our results, the astrocyte-microglia coexpression in the ZI seemed to increase after neuropathy-induced plasticity, as assessed with immunohistochemistry. CD68, a microglial marker in mammalian tissue, was used to identify microglia. In our results, CD68 expression levels were observed in the ZI of NP + Sham stim. rats. Coexpression of astrocytes and microglial cells was observed in the ZI area (Fig. 3). However, in the control and NP + MCS rats, we did not observe coexpression or plastic changes in astrocytes in the ZI area (Control, 100 ± 5.77; NP + Sham stim., 402.773 ± 23.39; NP + MCS, 111.36 ± 10.3). These results support the interpretation that neuropathic pain is associated with abnormal GFAP declines in the ZI, which are coupled with elevated CD68 expression.

**Figure 4:**
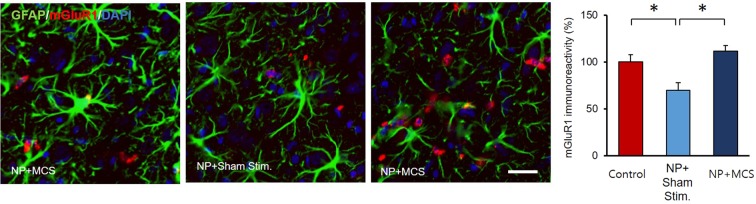
Comparison of GFAP-mGluR1 expression in the ZI following MCS. Immunohistochemical analysis of mGluR1 and GFAP expression, showing the effects of MCS. Double labeling revealed mGluR1 expression (red) in neurons in the ZI. MCS induced changes in mGluR1-positive spines, which appeared near astrocytes in the ZI. Intense mGluR1 expression was observed in NP + MCS rats. (Scale bars = 20 µm).

Glutamatergic neurotransmission is involved in most aspects of normal brain function, and the presence of metabotropic glutamate receptor 1 (mGluR1) indicates synaptic plasticity. In order to identify the expression of glutamatergic transmission, mGluR1 and GFAP were examined. In NP + MCS rats, the intense mGluR1 expression near astrocytes in the ZI demonstrated that highly activated neurons interacted with glial cells in the ZI (Control, 100 ± 7.82; NP + Sham stim., 70.26 ± 8.14; NP + MCS, 111.67 ± 6.21; Fig. 4). Figure 4 shows a neuronal-glial synapse in the ZI of an NP + MCS rat.

### Specificity of MCS effects

**Figure 5:**
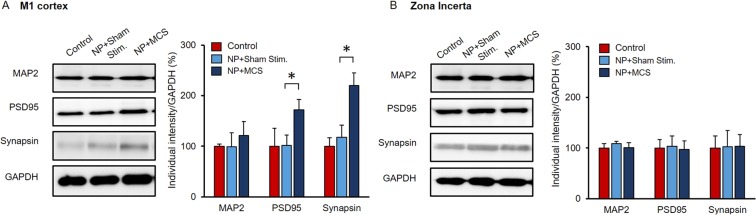
Expression levels of synaptic molecules after MCS. (**A**) Comparison of synaptic molecule expression in the M1 (normalized to GAPDH). PSD95 and synapsin levels in the M1 were significantly increased (*p < *0.05). Bar graph indicates the results of western blot analysis. In NP + Sham stim. rats, MAP2 level was 100 ± 4.7, PSD95 level was 100 ± 35, and synapsin level was 100 ± 14. However, in NP + MCS group, PSD95 (223.75 ± 26.85) and synapsin (219 ± 24.11) levels were significantly increased. MAP2 level was 136.08 ± 31.3. (**B**) Comparison of synaptic molecule expression in the ZI, showing no significant changes. In NP + Sham stim. rats and NP + MCS rats, MAP2 levels were 100 ± 8.5 and 126.46 ± 5.94, respectively; PSD95 levels were 100 ± 16 and 92.23 ± 19.36, respectively; and synapsin levels were 100 ± 23 and 106.07 ± 32.88, respectively *(*all *p* < 0.05*)*. Punctured M1 and ZI tissues were subjected to western blotting with antibodies against MAP2, PSD95, synapsin, and GAPDH antibodies. Full-length blots are presented in Supplementary Fig. S2.

Based on the abovementioned results, we hypothesized that increased mGluR expression in neuronal-glial synapses was related to repetitive MCS, and that increased tripartite complexes would lead to the generation of neurons and synapses. To test this hypothesis, we measured the protein expression levels of several major molecules related to synaptic plasticity, including MAP2, PSD95, and synapsin, in the M1 cortex and ZI area (Fig. 5A). The levels of these three proteins were upregulated in the M1 of NP + MCS rats (n = 3) compared to the M1 of NP + Sham stim. rats (n = 3). Protein expression levels were also measured in the ZI; however, MAP2, PSD95, and synapsin levels were not significantly increased in that area (Fig. 5B*, p* > 0.05).

## Discussion

We evaluated the changes in chronic NP-induced neural plasticity following MCS by assessing behavior, morphology, and local synaptic protein expression. As in our previous studies, peripheral nerve injury induced significant hind limb hyperalgesia, as evidenced by lowered withdrawal thresholds^14^. In addition, we observed an increased mechanical threshold after repetitive MCS, and found that NP decreased GFAP expression in the ZI. The reduction in astrocyte expression in the the ZI of NP + Sham stim. rats were consistent with maladaptive incertothalamic neuronal changes^10^. The restoration of GFAP expression following MCS suggests that protein expression by astrocytes is involved in pain modulation and synaptic plasticity. Furthermore, the increased expressions in glial cells and mGluR1 suggest alteration of synaptic plasticity in the ZI.

### Changes in astrocyte distribution in the ZI

Astrocytes are the most abundant cell type found in the brain^15^. They have intricate morphological interactions with neurons, making them well-positioned to influence synaptic transmission. Fine astrocytic processes ensheathe neuronal synapses, forming a tripartite complex with pre- and post-synaptic neuronal components^16^. The traditionally recognized role of astrocytes was macrophagic activity or metabolic support^17–19^. However, more recent studies have led to reconsideration of their functions^20,21^. Astrocytes are now thought to play several active roles in the brain, including the secretion and absorption of neural transmitters and the maintenance of blood–brain barrier^21–23^.

Although current evidence from animal and human studies suggests pain-modulating effects of MCS, the reason for variation in astrocyte numbers following MCS remains to be elucidated^13,24,25^. Several hypotheses have been proposed to explain how MCS reduces pain^14,26^. Previous studies showed that MCS stimulates serotonin secretion in the periaqueductal gray, either directly or indirectly, via the ZI^27,28^. In addition, serotonin reactivity in the spinal cord indicates that MCS appears to be a descending modulator of NP^28–30^. We hypothesized that MCS induces plasticity in the corticothalamic pathway, modulating synapses in the ZI. Our findings demonstrate a correlation between GFAP expression and the analgesic effect of MCS in a model of NP.

The effects of MCS treatments on behavioral parameters confirm that it decreases pain after nerve injury. After repetitive MCS sessions, astrocytic protein expression was restored. This suggests that the analgesic effects of MCS might persist due to joint neuronal and glial changes in the brain^14,31^, as the effect of MCS on pain may be related to astroglial changes in the ZI.

Microglia exhibited a strong increase in the expression of the microglia-specific marker CD68 in the ZI of NP + Sham stim. group. However, after the repetitive MCS period, protein levels of CD68 were reduced back to control levels. Similar results were observed in another study that applied tDCS to the primary motor cortex^32^. Although it is possible to deduce that protein expression by microglia is related to the synaptic changes in the ZI^33^, the relationship between the interaction of microglial activation and MCS-induced neuroplasticity is still remains controversial and is largely under investigation.

MCS induces a sudden change or interruption in the network status – either physiological or pathological^34^. Several studies showing increases or decreases in neuronal activity after MCS suggest that neuronal network reorganization occurs^35–37^. Therefore, it is also suspected that the MCS-induced modulation in the ZI is due to large-scale astrocytic activation representative of a new global status^34^. Cortical electric stimulation can directly activate astrocytes, which impacts neuronal signaling^38,39^. ATP released from astrocytes is degraded in the extracellular space by ecto-ATPase to adenosine, which accumulates in the extracellular space around the electrode; adenosine is thought to be a key mediator promoting the efficacy of electric stimulation in suppressing abnormal neuronal activity^39^. Agnesi *et al*.^40^ reported that MCS produced analgesic effects concomitant with glutamate and adenosine release.

It remains unproven whether local synaptic modulation in astrocytic processes can extend to activate the whole astrocyte, whereby multiple synapses in different areas could be modulated, either uniformly or differentially, allowing astrocytes to function as a network modulator.

### Regulation of synaptic plasticity

Neuroplasticity is believed to be fundamental to MCS efficacy^41^. In this study, we showed significant changes in astrocytes in the ZI, which is responsible for NP modulation. In this study, to identify the MCS-induced neuronal change, we chose the ZI region and analyzed the expression levels of proteins. Concerning the regulation of nociceptive processing in the thalamus, the ZI (GABAergic nucleus located in the diencephalon) inhibits the flow of nociceptive and somatosensory information in the posterior thalamus. We hypothesized that the expression of astrocytes changed after NP and NP with MCS. For the comparison, we divided the animals into three groups (sham-operated control, NP + MCS, and NP + Sham stimulation). Our western blot results show a change in the neuroglia in the ZI after neuropathic pain. In addition, when NP was followed by MCS, the reduced astrocytic protein expression recovered to the same value as the control (sham injury). The increase in GFAP expression after MCS and the structural changes in neuronal-glial synapses indicate that treatment induced significant plastic changes in the ZI. Regarding the relationship between synaptogenesis and the increase in astrocyte number, this occurred only in the M1 cortex; there were no significant changes in synaptic protein expression in the ZI. These results suggest that epidural electrical stimulation induced the formation of new synapses in the M1 cortex, whereas the ZI produced astrocyte-induced pain control rather than synaptogenesis.

Structural neuronal changes occur in synapses in abnormal pain states, whereby astrocytes modulate signal processing and integration via complex neuronal–glial networks. This arrangement means that both astrocytes and neurons are likely to be affected by electrical stimulation^42,43^. These processes directly involve N-methyl-D-aspartate receptors (NMDARs) as well as specific gliotransmitters^44^. Astrocytes modulate NMDAR activity by releasing glutamate and providing the coagonist D-serine, which, similar to glycine, acts on distinct NMDAR populations^44^. Astrocyte-derived D-serine modulates NMDAR currents and is necessary for synaptic plasticity, as long-term potentiation does not occur in the absence of astrocytes but can be induced with exogenous D-serine^45–47^. Recent results suggest that D-serine release can be dynamic and provides the potential for astrocytes to induce rapid changes in NMDAR currents^48–50^.

### Involvement of astrocytes in synaptic plasticity after MCS

We studied potential factors influencing structural plasticity in astrocytes after repetitive MCS. We previously reported MCS-induced pain modulation in NP rats, as well as altered glial-neuronal interactions and morphological changes after nerve injury^14^. Coupled with our present results, the existing evidence supports a glial-neuronal interaction mechanism of pain pathogenesis.

Panatier *et al*.^51,52^ observed that astrocytic processes detect local synaptic activity (neurotransmitter release), which causes a local Ca^2+^-evoked response mediated through metabotropic glutamate subtype 5 receptor (mGluR5) activation. The literature suggests that such glial modulation may adjust synaptic efficacy so that it can conform to various plasticity events in specific brain regions, as adenosine release can either increase transmitter release (via presynaptic adenosine A_2A_ receptor activation) or decrease it (via adenosine A_1_ receptor activation). Since astrocytes have very complex spatial relationships with adjacent neurons and maintain several types of synapses, it is possible to use different transmitters to interact with these structures^53–55^.

Several hypotheses have been proposed to explain how MCS ameliorates pain^13,56–58^. Here, we propose that MCS modulates synaptic strength by affecting astrocytes in the ZI, leading to synaptic plasticity. As astrocyte processes are ideally located in apposition with pre- and post-synaptic neuronal elements throughout the CNS, astrocytes likely regulate basal synaptic transmission and plasticity in the brain^59,60^. We observed decreased GFAP expression in the ZI after nerve injury, but expression was restored after MCS. In our results, intense mGluR1 expressions were observed in NP + MCS rats. This could demonstrate that when presynaptic neuron releases glutamate, the neurotransmitter binds to receptors on the postsynaptic neuron, signaling the latter neuron to be activated. This glutamate release is associated with a corresponding increase in calcium levels in the adjacent neurons. Modulating calcium levels could influence neural signaling patterns. The astrocytes wrap around synapses, and they seem perfectly placed to play an active role in influencing the communication between neurons. Neuronal activity can directly affect calcium levels in neighboring astrocytes, after which the astrocytes can complete the cycle by signaling back to the neurons through glutamate release^19,20,43^.

In the present study, we observed MCS-induced pain modulation in NP rats and morphological changes of astrocytes. These results support astrocyte expression changes as a mechanism of pain modulation. Understanding how astrocytic-neuronal relationships change after MCS in different cortical areas will clarify our understanding of pain modulation. Further knowledge of maladaptive synaptic changes would be helpful for designing treatments to control pain.

## Methods

### Animals and design overview

All animal experiments in this study were performed in accordance with the guidelines for the ethical use of conscious animals in pain research published by the International Association for the Study of Pain, and were approved by the Institutional Animal Care and Use Committee of Yonsei University Health System (protocol number 2016–0061). Male Sprague Dawley rats (N = 36, 9 weeks old, 200–220 g; Koatech, Pyeongtaek, Korea) were housed in ventilated plastic cages (3 /cage) with soft bedding, and were maintained on a 12-/12-hour light/dark cycle (lights on at 07:00) at a constant temperature (22 ± 2 °C) and humidity (50 ± 10%) in an animal facility accredited by AAALAC International. The rats were divided into three groups. The animals were fed standard rat chow and had access to tap water *ad libitum*.

### Animal model of NP

The rats were anesthetized with intraperitoneal (i.p.) sodium pentobarbital (50 mg/kg). The respiratory rate, corneal reflex, and tail-pinch response were monitored to ensure that the animals were sufficiently anesthetized. The surgical procedure and MCS setup were performed as we described previously^14^. Briefly, a segment of the sciatic nerve was exposed, and three divisions of the sciatic nerve (namely, the common peroneal, tibial, and sural nerves) were clearly separated. The tibial and sural nerves were ligated and transected, but the common peroneal nerve was left intact. Complete hemostasis was confirmed, and the wound was closed with muscle and skin sutures. Sham control group underwent the same operation without any nerve damage. All efforts were made to minimize animal suffering and reduce the number of animals used.

### Stimulation electrode implantation

Two weeks after NP surgery, the animals were deeply anesthetized and placed on a thermoregulated heating pad in a stereotaxic frame. A local anesthetic (2% lidocaine) was applied to the incision sites 5 min before the surgery. A hole was made in the skull overlying the M1, and custom-made insulated bipolar platinum electrodes (height, 70 μm; exposed tip, 50 μm; distance between electrodes, 500 μm) were applied epidurally above the M1 at stereotaxic coordinates determined from previous MCS experiments (anterior, 1.8 mm; lateral, 2 mm), contralateral to the nerve injury site (Fig. 1A)^61^. The stimulation electrodes for MCS were held in place with two bone screws and dental cement.

### Behavioral testing

Changes in the mechanical paw withdrawal threshold were measured at 1, 4, 7, and 14 days after nerve or sham surgery. Rats were habituated for 10 min to the testing cages, which consisted of metal mesh floors under plastic domes. Mechanical allodynia was measured by assessing thresholds for hind paw withdrawal upon stimulation with an electrically controlled von Frey filament (Ugo Basile, Varese, Italy). Licking or rapid withdrawal of the hind paw was considered a positive response. Mechanical forces were recorded for each withdrawal. The responses were measured seven times, and the means were calculated after the maximum and minimum values were excluded. Nociceptive tests were performed after MCS in awake rats for 10 days. Electrodes were connected to a stimulator (A385, WPI, Sarasota, FL, USA) for repetitive MCS. For MCS, the M1 of awake animals were stimulated for 30 min (continuously at 50 μA, 50 Hz, and 300 μs pulses). Sham MCS group underwent the same procedure without any electrical stimulation. After the last behavioral test, the animals were anesthetized with urethane (1.25 mg/kg, i.p.), and tissue was collected for western blot and immunohistochemistry assays.

### Western blot analysis

Rats were anesthetized and sacrificed prior to the ZI tissue collection (5 /group). The contralateral and ventrolateral ZI were quickly dissected, frozen on dry ice, and stored at −70 °C. For protein extraction, the samples of each group was pooled and homogenized with lysis buffer (ProPrep; Intron Biotechnology, Pyeongtaek, Korea) containing phosphatase inhibitors (Phosstop; Roche, Mannheim, Germany). The samples were centrifuged at 12,000 × *g* for 20 min at 4 °C, and then the supernatants were collected. Total protein concentrations were assessed with a spectrophotometer (Nano Drop ND-1000; Thermo Fisher Scientific, Waltham, MA, USA), and 30 mg of protein per well was denatured and run on 10% gels (Bio-Rad, Hercules, CA, USA). The proteins were transferred onto a polyvinylidene difluoride membrane (Merck Millipore, Darmstadt, Germany), and the membranes were blocked by incubation in 3% skim milk. The membranes were incubated with primary antibodies against glial fibrillary acidic protein (GFAP) (1:5000; catalog ab7260, Abcam, Cambridge, UK), NeuN (1:5000; catalog MAB377, Millipore), microtubule-associated protein 2 (MAP2) (1:1000; catalog ab11267, Abcam), postsynaptic density-95 (PSD95) (1:500; catalog ab18258, Abcam), synapsin (1:500; catalog ab64581, Abcam), CD68 (1:1000; cat MAB1435, Millipore), and β-actin (1:10,000; catalog #3700; Cell Signaling Technology) or glyceraldehyde 3-phosphate dehydrogenase (GAPDH) (1:10000; catalog #2118, Cell Signaling Technology, Danvers, MA, USA). The membranes were then incubated with the appropriate horseradish peroxidase-conjugated anti-rabbit or anti-mouse secondary antibodies (1:10,000; no. 7074 and 7076, Cell Signaling Technology). Protein bands were visualized by applying a chemiluminescent substrate (GE Healthcare, Little Chalfont, UK) and observed using LAS 4000 system (GE Healthcare). β-Actin and GAPDH were used as the loading controls. Immunoblotting experiments were replicated at least four times.

### Immunohistochemistry

Anesthetized rats (4 /group) were perfused with 300 ml 0.9% NaCl and 4% paraformaldehyde in phosphate-buffered saline (PBS), and the extracted brains were stored with 4% paraformaldehyde and then 30% sucrose for 48 hours before being frozen in an embedding compound. The frozen whole brains were cut to a thickness of 30 μm with a cryostat (HM525, Thermo Scientific). The section slides were incubated overnight at 4 °C with primary antibodies against GFAP (1:1000; catalog ab7260, Abcam), NeuN (1:1000; catalog MAB377, Millipore), MAP2 (1:1000; catalog ab11267, Abcam), PSD95 (1:500; catalog ab18258, Abcam), synapsin (1:500; catalog ab64581; Abcam), and CD68 (1:1000; cat MAB1435; Millipore), washed with PBS, and incubated for 2 hours at room temperature with Alexa Fluor 488 and Cy^tm^ 3-conjugated AffiniPure F(ab’)_2_ Fragment Donkey Anti-Mouse IgG secondary antibodies (1:1000; Jackson ImmunoResearch, West Grove, PA, USA). DAPI was used for counterstaining. Immunofluorescent sections were imaged by LSM700 confocal microscope (Zeiss, Oberkochen, Germany) using 10× and 40× PlanApo oil-immersion lens. Briefly, 12 μm-thick confocal Z-stacks of the synaptic zone in ZI were captured. Three image stacks per rat (4 /group) were used for analyses. The number of cells with colocalized GFAP and mGluR1 was quantified. Three images per rat (4 /group) were used for analyses.

### Statistical analysis

Behavioral test data were analyzed by two-way analysis of variance (ANOVA) followed by Dunnett’s post-hoc multiple comparison test to determine significance. One-way ANOVA followed by Dunnet’s post-hoc analysis was used in immunohistochemistry and western blotting data. All statistical analyses were performed with SPSS 22.0 software (IBM Corporation, Armonk, NY, USA). All values are expressed as the mean ± standard error of the mean (SEM). P-values less than 0.05 were considered statistically significant.

## Supplementary information

Supplementary Information.
