# Supplementary material for: Astroglial changes in the zona incerta in response to motor cortex stimulation in a rat model of chronic neuropathy
Source: Sci Rep. 2020 Jan 22;10:943. doi: 10.1038/s41598-020-57797-y (PMC6976635; doi:10.1038/s41598-020-57797-y)
Supplement: Supplementary file 1 — Supplementary Information. [file 41598_2020_57797_MOESM1_ESM.pdf]

Supplementary information

Astroglial changes in the zona incerta in response to motor cortex stimulation in a rat model of chronic neuropathy

Myeounghoon Cha[1], Kyung Hee Lee[2], Bae Hwan Lee[1,3*]

[1] Department of Physiology, Yonsei University College of Medicine, Seoul 03722, Republic of Korea
[2] Department of Dental Hygiene, Division of Health Science, Dongseo University, Busan 47011, Republic of Korea
[3] Brain Korea 21 PLUS Project for Medical Science, Brain Research Institute, Epilepsy Research Institute, Yonsei University College of Medicine, Seoul 03722, Republic of Korea

* Corresponding Author
Bae Hwan Lee, PhD
Department of Physiology, Yonsei University College of Medicine
50-1, Yonsei-ro, Seodaemun-gu
Seoul 03722, Korea
Tel.: +82.2.2228.1711
Fax: +82.2.393.0203
E-mail address: bhlee@yuhs.ac

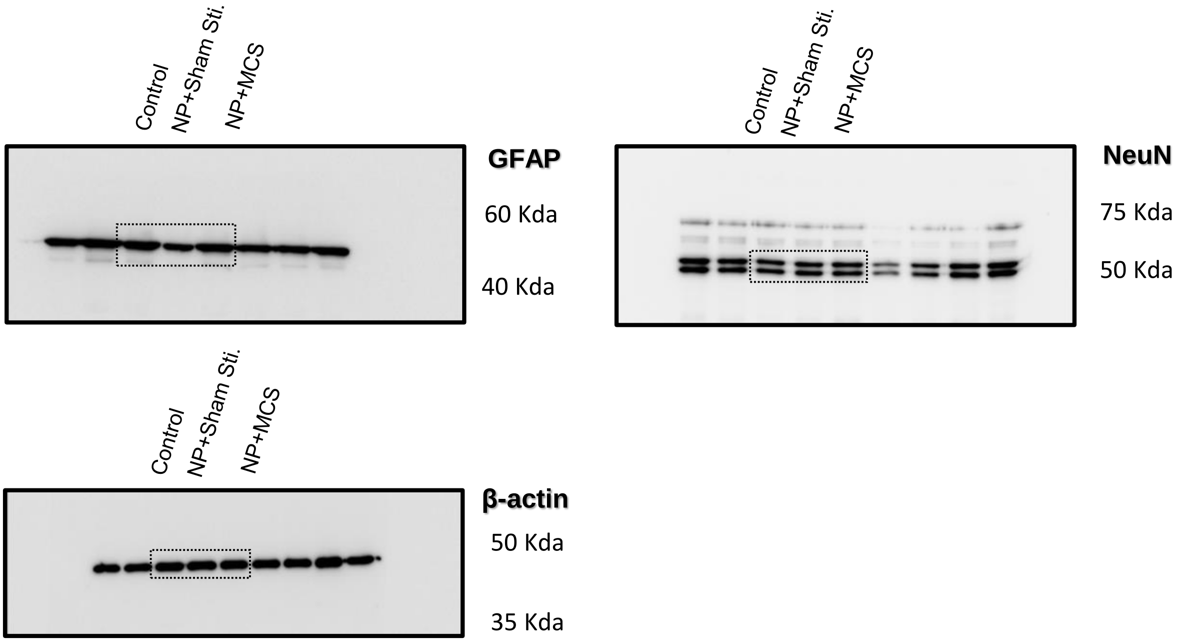

Supplementary Figure S1. Full-length blots of western blotting shown in Figure 2E. Punctured the ZI tissues were subjected to western blotting with GFAP or NeuN antibodies. Anti-β-actin antibody was used as a loading control. The cropped parts indicate by the gray dashed lines are shown in Figure 2E

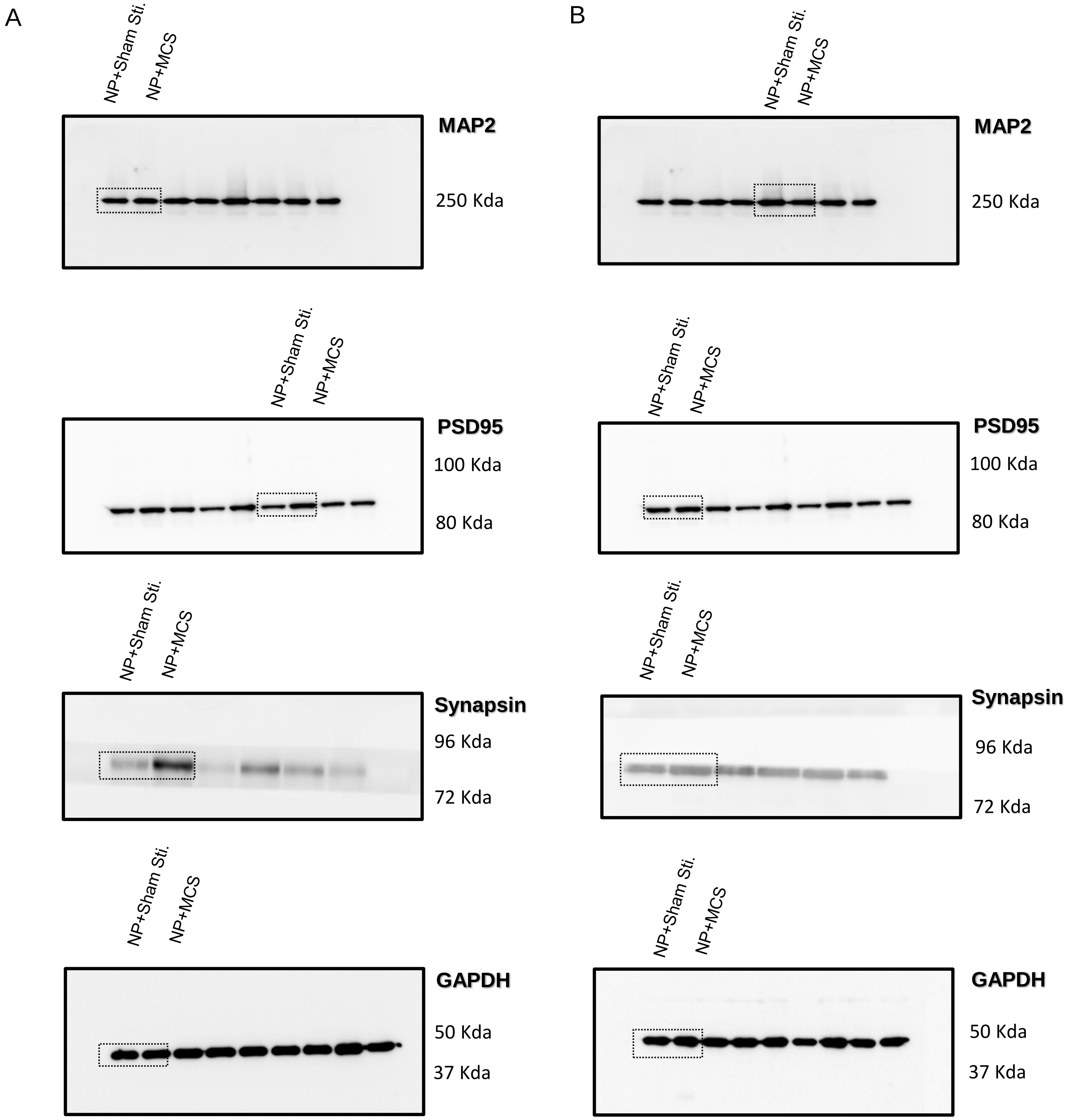

Supplementary Figure S2. Full-length blots of western blotting shown in Figure 5A and B. Punctured the M1 and ZI tissues were subjected to western blotting with MAP2, PSD95, and Synapsin antibodies. Anti-GAPDH antibody was used as a loading control. The cropped parts indicate by the gray dashed lines are shown in Figure 5A and B.
